# Tree–shrub–grass composite woodland better facilitates emotional recovery in college students emotion better than other plant communities

**DOI:** 10.3389/fpsyg.2024.1285792

**Published:** 2024-01-29

**Authors:** Wen Jun Fu, Fei Gao, Xing Zhang, Bo Dong, Xi Lin Chen, Xin Xu, Zhi Yu Yang, Yong Liu

**Affiliations:** ^1^School of Architecture and Urban Planning, Suzhou University of Science and Technology, Suzhou, Jiangsu, China; ^2^School of Education, Suzhou University of Science and Technology, Suzhou, Jiangsu, China; ^3^Suqian High Speed Railway Construction and Development Co., Ltd., Suqian, Jiangsu, China

**Keywords:** plant community, restorative landscape, positive emotions, negative emotions, EEG

## Abstract

Previous research has indicated that natural landscapes exhibit a greater capacity for ameliorating negative emotional states in individuals when compared to urban landscapes. Nevertheless, significant scientific inquiries, such as the uniformity of the rejuvenating effect across distinct categories of natural landscapes on college students and the choice of the optimal plant community for achieving the most potent restorative effect, remain unexplored. This study aimed to address these questions by selecting four plant communities (single-layer grassland, single-layer woodland, tree-grass composite woodland, tree-shrub-grass composite woodland) and using an electroencephalography method to capture the neuroelectric activity of the participants in combination with the Positive and Negative Affect Schedule score to explore the effects of plant community types on emotional recovery. The results showed that all four plant communities significantly increased positive emotions and significantly reduced negative emotions. There was no significant difference in the recovery effect of positive emotions among the four plant community types, but there was a significant difference in the recovery effect of negative emotions. The effect of tree-shrub-grass composite woodland on the negative emotion recovery effect is the best; the EEG results found that the alpha wave amplitude induced by the tree-shrub-grass composite woodland was significantly higher than that of the other three groups of plant communities, and the EEG and behavioral results were consistent. The results show that the tree-shrub-grass composite woodland has the best restoration effect and has stronger planning and design significance.

## 1 Introduction

Due to the rapid development of urbanization, the high-intensity pace of life, study pressure, employment pressure, and indifferent urban environment, people’s chances for contact with the natural environment are gradually decreasing (Zhao et al., 2022). Some research shows that college students in particular have mental health problems (Urena, 2023). With the expansion of college enrollment and the intensification of academic pressure of higher education, the requirements for college students’ psychological quality and adaptability have become increasingly critical; especially given the fierce social competition and severe employment situation, college students’ mental health problems are on the rise (Zhang, 2022). However, not only is the mental health of college students related to their personal development—it also has a profound impact on the stable development of families, schools, and the entire society. Therefore, the mental health problems of college students should receive attention from researchers, and the construction of restorative landscapes should be further promoted to gain an in-depth understanding of college students’ emotional perception and restorative impact mechanisms according to different landscape environments.

As an important part of human psychology, emotions produce corresponding emotional experiences according to changes in the situation during interactions between people and situations (Fu, 2016). Moreover, in the same environment, interactions at different times will also produce different emotional experiences (Zhang et al., 2023). When people are in different emotional states, there are differences in their perception and evaluation of their environment (Ibrahim et al., 2021). An increase in positive emotions and a decrease in negative emotions are helpful for people’s healthy recovery (McNeil and Repetti, 2022). Positive emotions help improve cognitive abilities, establish a state of positive thinking (Li, 2015), promote top-down intuitive processing, and improve creativity (Ye et al., 2023). At the same time, the existence of good and stable emotions is conducive to increasing college students’ resistance to the negative influence of the social environment. Therefore, regulating one’s own negative emotions is of great significance to the physical and mental health of college students (Guo et al., 2023).

Emotional recovery is closely related to the landscape environment. In landscape environment design, a good and comfortable natural environment (Ulrich, 1984), green space (Ulrich, 1993), or landscape facilities (Deng et al., 2020) are considered to have the ability to promote emotional recovery, reduce stress, and increase awareness-boosting features. Contact with nature in an urban environment can achieve the purpose of psychological recovery (Maas et al., 2006), and plant communities with soft and attractive effects are more prominent for emotional recovery (Liu and Li, 2020). Research shows that experiencing natural environments is an effective route for emotional recovery and helps to improve negative emotions (Yu et al., 2018; Furuyashiki et al., 2019). For example, gardening activities have a significant effect on negative emotions (Meore et al., 2021). And exercising in the forest environment can reduce negative emotions to a greater extent (Mitchell, 2013). At the same time, humans stay in a natural environment for a long time have better mental health (Beyer et al., 2014).

Previous studies contend that “the natural environment is more restorative than the artificial environment” (Bowler et al., 2010) and “the more natural elements, the higher the restorative effect” (Methorst et al., 2021). Moreover, “different types of natural landscapes have different restorative properties” (Sonntag-Öström et al., 2015). However, a systematic understanding of the characteristics of urban green environments related to psychological recovery persists (Karmanov and Hamel, 2008). Therefore, building emotionally restorative urban parks remains a challenge. As an important component of the landscape environment, plant communities have an important impact on the comfort and emotional recovery of human settlements (Jianbin et al., 2021). Few studies to date have focused on “what types of plants have better landscape-restoration properties”; however, deciding which type of plants to use in landscape design is extremely important. Thus, the present study is the first to explore “the restorative properties of different types of plants” to provide insights into landscape design and provide theoretical support.

As a physiological function, visual perception relies on subjective images and uses visual senses and information-processing organs to process and analyze information (Rogers, 2011), thereby understanding the objective world (Spence, 2020). Among the five human perceptions, 80–90% of sensory stimulation comes from visual perception (Chen et al., 2016). In the cognitive system, visual stimulation takes priority over consciousness (Sklar et al., 2021). At the same time, good visual perception can further promote people’s emotional recovery and reduce negative emotions. Therefore, some scholars have proposed relevant visual-optimization design strategies for coastal buildings based on visual perception (Hu and Chen, 2020) or analyzed the visual characteristics of greenways and proposed corresponding planning and designs for greenway landscapes (Pan and Jiang, 2022). At the same time, existing research has shown that visual perception plays a certain role in emotional recovery. Viewing natural scenes, such as open lawns, diverse vegetation, and bright flowers, can soothe people’s emotions through visual perception (He et al., 2022). Therefore, by analyzing people’s preferences and characteristics in visual perception, we can further promote the construction of a more comfortable and pleasant landscape environment and promote the healthy development of people’s physical and mental health.

Previous studies have mostly employed self-report questionnaires (Lee et al., 2022), which relied on subjects’ perceptions of the current state at the conscious level. With this approach, however, there was inevitable “subjectivity” and insufficient objectivity. In recent years, biofeedback technology has become an important means by which to study the relationship between humans and nature. Because electroencephalography (EEG) can objectively describe a person’s mental state, it has the advantages of being intuitive and highly manipulable (Knaust et al., 2022). Current research using EEG technology has mostly focused on experiences with nature (Chen et al., 2016), the frequency of viewing plants/greenery (Nie et al., 2022), plant morphology preferences (Ding et al., 2022), and other aspects. To date, a few studies have used EEG to study whether there are differences in the effects of different plant communities on mood recovery. Some literature suggests that people with different social backgrounds and ages will evaluate visual landscapes differently (Sevenant and Antrop, 2010). However, among the many current studies examining the impact of the environment on people’s physical and mental health, few environmental studies have examined this phenomenon among college students. The present study focuses explicitly on Chinese college students. According to our experimental design, college students were used as the experimental population to reduce differences among the experimental groups. Taking the effect of plant communities on emotional recovery as the research purpose, we explored which plant community has the best effect on college students’ emotional recovery under visual perception. This study combined a subjective questionnaire and EEG data to objectively verify the difference in the effect of different plant communities on the emotional recovery of college students, which can provide scientific method guidance for plant community planning of urban parks in the future.

## 2 Materials and methods

### 2.1 Stimulus materials

According to the diversity of plant species in Suzhou City, Huqiu Wetland Park was selected as the sample site after investigating and screening several parks in Suzhou. Tiger Hill Wetland Park is located in northwest downtown Suzhou, Jiangsu Province, spanning Gusu and Xiangcheng Districts. It serves as an important component of Suzhou’s green space system and ecological civilization construction, acting as a “green wedge” and “ecological green lung.” The park exhibits diverse plant communities and a favorable ecological environment, rendering it a good representative of similar parks. Initial surveys of park plants indicate that single-layer grassland, single-layer woodland, tree–grass composite woodland, and tree–shrub–grass composite woodland are common and widely used in parks, while shrub–grass composite woodland is used less commonly. The common plant species in Suzhou were selected based on the criteria of plant safety, easy access and similar plant characteristics. Second, in order to avoid the impact of different colors on the subjects, plant species with similar tones were selected. Finally, when selecting a sample plot, we aimed to select a plant community with a basically consistent plant composition. Consequently, the plant communities studied in this experiment included singlelayer grassland (*Festuca elata* Keng ex E. B. Alexeev), single-layer woodland [*Taxodium distichum* (L.) Rich.], tree–grass composite woodland [*Osmanthus fragrans* (Thunb.) Lour. and *Festuca elata* Keng ex E. B. Alexeev], and tree–shrub–grass composite woodland [*Celtis sinensis* Pers., *Rhododendron* × *pulchrum Sweet and Festuca elata* Keng ex E. B. Alexeev].

**Figure 1:**
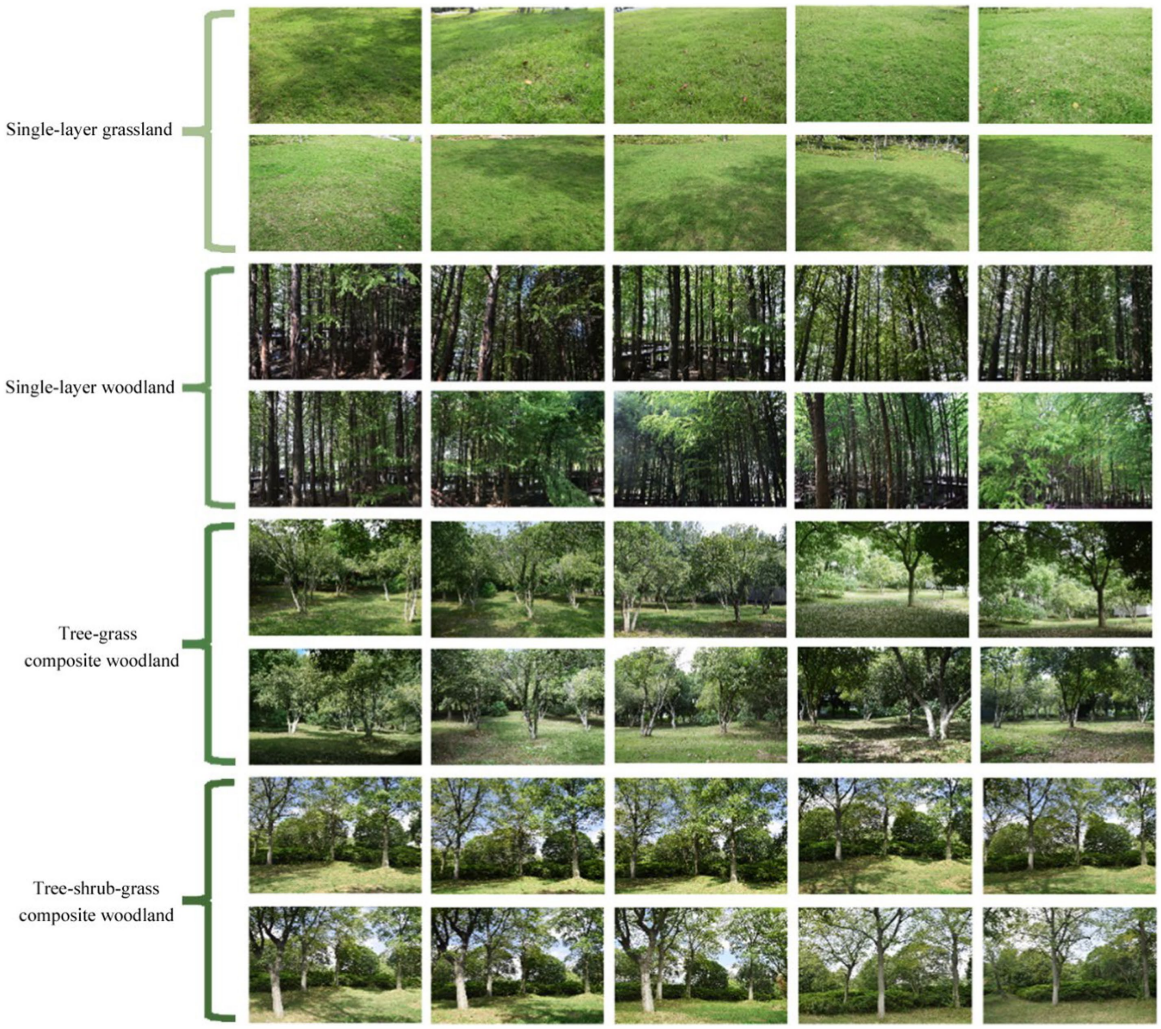
Two-dimensional color photographs of the selected study area.

The EEG experiment in this study employed photographs of different plant communities as experimental stimuli. After conducting several visits and investigations in Huqiu Wetland Park, to minimize experimental errors, a digital camera (Nikon D7100; Nikon Corporation, Tokyo, Japan) was used for photography in this study. The camera settings, including aperture, shutter speed, and other parameters, were selected in automatic mode, and the shooting time was standardized from 10:00 am to 1:00 pm, spanning October 1, 2022, to October 23, 2022. The photography sessions took place under clear weather conditions and without wind interference. More than 600 photos were captured from various angles of the plant communities and screened to ensure they met the criteria of a green overall tone and exhibited healthy plant growth. Ten photos were selected for each plant community type, taken from various shooting angles, resulting in a total of 40 photos (Figure 1). The selected photos were processed for details using Photoshop Creative Cloud 2018 (Adobe Inc., San Jose, CA, United States) to ensure uniformity.

Visual stimulation materials were presented using E-prime software. The size of each photo in this experiment was 6,000 × 4,000 pixels. For display, we used an XPS 8930 Dell computer; the size of the monitor (G2722HS) was 27 inches, and the resolution was 1,920 × 1,080 pixels.

### 2.2 Participants

The study sample size was determined using the G*Power 3.1 software. The class I error probability (α error probability) was set to 0.05, the test power (1 − β error probability) was set to 0.8, and a medium effect size (*f* = 0.25) was assumed. The calculated sample size was 24; thus, a total of 24 students from Suzhou University of Science and Technology in Jiangsu Province aged 18–25 years old were recruited for the study, including nine male individuals and 15 female individuals. All participants were right-handed, had normal hearing and vision or corrected vision, had no history of neurological or psychiatric disorders, and had not experienced any brain injuries. Participants received compensation at the end of the experiment. Additionally, participants were instructed to abstain from consuming coffee, alcohol, and other stimulant drinks within 24 h prior to the experiment to eliminate the potential effects of caffeine and alcohol and ensure sufficient sleep so as to avoid any abnormal EEG signals. Prior to the start of the experiment, participants were provided with a detailed description of the research. A total of 24 data points were collected during this study, with four subjects being excluded due to abnormal EEG signals and abnormal scale results. Therefore, the final dataset consisted of 20 valid experimental data points.

### 2.3 Experimental procedure

The EEG laboratory environment was a closed and quiet space designed to minimize external noise and other factors that could disturb the subjects to ensure the accuracy of the EEG data. In the non-experimental phase, the laboratory is in the ventilation phase to avoid the presence of peculiar smells that affect the subject’s perception. During the experiment, subjects were instructed to maintain a steady sitting position and to avoid head movements or swallowing saliva to prevent any interference with the collection of EEG signals. Visual stimuli consisted of four photos, each representing a different plant community, as follows: single-layer grassland, single-layer woodland, tree–grass composite woodland, and tree–shrub–grass composite woodland.

Each group participated in two stages of the experiment: pre-recovery (P_1_) and post-recovery (P_2_). The pre-recovery stage (P_1_) involved 3 min of stress stimulation, which included solving mathematical calculation problems (Duan and Li, 2022) and exposure to harsh audio (de Kort et al., 2006). In this stage, harsh audio with a background sound level of 40–50 dB was used, and subjects were required to accurately solve 30 mathematical problems within 3 min, effectively inducing pressure (Markus et al., 2000). Additionally, participants completed the Positive and Negative Affect Schedule (PANAS) questionnaire during this stage.

**Figure 2:**
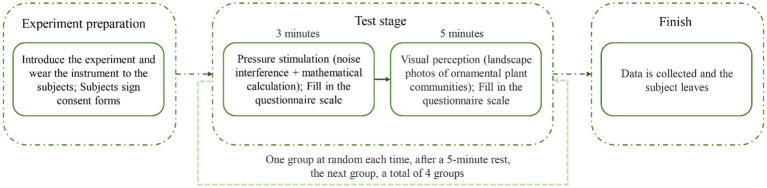
Steps of visual perception experiment.

In the post-recovery phase (P_2_), subjects viewed a set of plant community photos for 5 min. The photos were presented in a random order, with each photo displayed for 30 s. Subjects also completed the PANAS questionnaire again. After completing one round of the experiment, subjects were given a 5-min rest period to eliminate any influence from the previous round and to allow their emotions to calm down before proceeding to the next round of the experiment. The entire experiment lasted approximately 60 min (Figure 2).

### 2.4 Data extraction

Data collection was conducted using 62 Ag/AgCl electrodes of the NeuroScan SynAmps2 system with a resistance of less than 7 kΩ and a sampling rate of 500 Hz. The online reference was the left mastoid process. It is known that EEG signals can objectively reflect the emotional state of subjects. During EEG recording, electrical signals from the cerebral cortex are recorded as brain waves and can be categorized into the following four bands: delta waves (0.5–3 Hz), theta waves (4–7 Hz), alpha waves (8–13 Hz), and beta waves (14–30 Hz) (Oh et al., 2019). Alpha waves are present when the brain is awake and relaxed and enhanced during happy or meditative states, while the other three bands appear during sleep, fatigue, tension, and anxiety. In this experiment, alpha waves were analyzed to evaluate the effect of emotional recovery following exposure to four plant communities in college students under visual perception conditions.

Positive and negative emotions are important components of mental health. In this study, PANAS was used to measure the subjective emotional changes of subjects. PANAS consists of 20 emotion words, with 10 words assigned for each emotion category, and has high credibility (Watson et al., 1988). After visual perception, higher positive emotion scores and lower negative emotion scores indicate better emotional recovery. PANAS uses a 5-point Likert scale, with 1 point indicating very little or none and 5 points indicating very much.

## 3 Data analysis

The questionnaire data in this study were processed using SPSS Statistics 26.0 (IBM Corporation, Armonk, NY, USA), and the formulae used for calculating the emotional change value were as follows:

$$\begin{array}{l} \mathrm{Negative}\;\mathrm{emotion}\;\mathrm{change}\;\mathrm{value}\;\left(\Delta P_{NA}\right):\\ \Delta P_{NA}=\left(P_{1-NA}-P_{2-NA}\right)/P_{1-NA}\text{.} \end{array}$$

$$\begin{array}{l} \mathrm{Positive}\;\mathrm{mood}\;\mathrm{change}\;\mathrm{value}\;\left(\Delta P_{PA}\right):\\ \Delta P_{PA}=\left(P_{1-PA}-P_{2-PA}\right)/P_{1-PA}\text{.} \end{array}$$

where P is the mean value of emotional words, P_1 − NA_ is the mean value of negative emotional words before recovery, P_2 − NA_ is the mean value of negative emotional words after recovery, P_1 − PA_ is the mean value of positive emotional words before recovery, P_2 − PA_ is the mean value of positive emotional words after recovery, and ΔP is the change value of emotional words. A positive Δ*p* value indicates that the mean value of emotional words before recovery is greater than the mean value after recovery; otherwise, it indicates that the mean value of emotional words after recovery is greater than that before recovery.

The PA and NA values in the pre-recovery (P_1_) stage were expected to be significantly different from those in the post-recovery (P_2_) stage. In order to determine whether each type of plant community elicited psychological changes, a paired-samples *t* test was used to compare PA values and NA values in the two stages before recovery (P_1_) and after recovery (P_2_) to test the effectiveness of stressors. We then used repeated-measures analysis of variance in a general linear model to compare the differences in emotional recovery effects between different plant communities.

By using Matlab R2022b (MathWorks, Natick, MA, United States) to analyze the EEG data in the process of visual perception, the difference in emotional-recovery effect between different plant communities under objective conditions was compared. The collected EEG signals in a CDT-type file format were converted to a .set file format using the EEGLAB toolkit; then, the data were processed and analyzed by Fieldtrip (Oostenveld et al., 2011). First, the electrode positioning was confirmed, and the re-reference was set as the whole brain average; then, high–low pass filtering of 1–40 Hz and baseline correction of −1 to 0 Hz were performed. Blink and eye movement artifacts were manually identified and removed by independent component analysisto complete EEG data preprocessing (Nie et al., 2022). For ERP analysis, the continuous EEG signals were divided into 29,000-ms segments (encompassing 1,000 ms before stimulation to 28,000 ms after stimulation). For frequency analysis, 1–40 Hz was selected for power spectrum conversion.

## 4 Results

### 4.1 Results of questionnaire scale data analysis

#### 4.1.1 Positive and negative mood changes before and after recovery

In the pre-recovery (P_1_) stage, the default plant landscape type had no effect on the subjects’ positive and negative emotions. The NA scores of single-layer grassland, tree–grass composite woodland, tree–shrub–grass composite woodland, and single-layer woodland in the pre-restoration (P_1_) stage were analyzed by variance analysis, and the obtained results indicated that the four plant communities induced the same negative emotions in the pre-recovery (P_1_) stage [*F* (3, 57) = 1.69]. Similarly, the PA scores of single-layer grassland, tree–grass composite woodland, tree–shrub–grass composite woodland, and single-layer woodland in the pre-recovery (P_1_) stage were analyzed by variance analysis, and the results show that [*F* (3, 57) = 0.63]. indicated that in the pre-recovery (P_1_) stage of four plant communities, all plant communities induced the same positive emotions (Table 1). In summary, there was no significant difference in stress levels between the groups in the pre-community recovery (P_1_) stage. Therefore, the differences between physiological and psychological states were later analyzed and attributed to the visual perception of different plant communities.

**Table 1:** Results of PA and NA analysis after exposure to stressors.

P_1_-PANA score	Plant communities	M	SD	*F*	*p*	*η*^2^
NA score	Single-layer grassland	21.20	8.95	1.69	0.179	0.082
	Tree-grass composite woodland	22.10	9.11			
	Tree-shrub-grass composite woodland	24.75	6.77			
	Single-layer woodland	22.90	9.74			
PA score	Single-layer grassland	21.90	8.49	0.63	0.597	0.032
	Tree-grass composite woodland	20.75	7.48			
	Tree-shrub-grass composite woodland	22.45	8.65			
	Single-layer woodland	20.80	6.45			

*p < 0.050, **p < 0.010, ***p < 0.001. *N* = 20 for each group.

**Figure 3:**
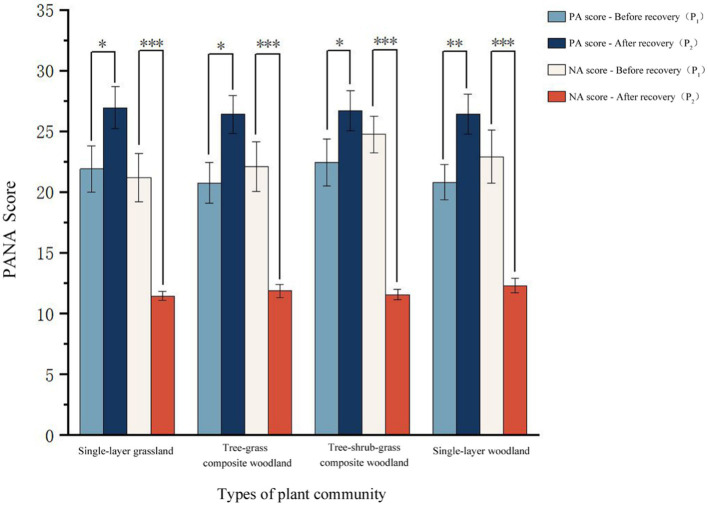
Comparison of the difference of scores before (P_1_) and after (P_2_) PANA restoration in four groups of plant community landscape(*p < 0.050, **p < 0.010, ***p < 0.001); Comparison of the di; Data is expressed as the *M*, and *N* = 20 for each group.

The PA and NA scores of the four groups of plant communities before (P_1_) and after (P_2_) restoration were tested by paired-samples *t* test. The results showed that the NA scores of all subjects decreased after viewing the four groups of photos, indicating that the negative emotions of the subjects were alleviated in the stage of recovery (P_2_). At the same time, their PA scores all increased, indicating that the positive emotions of the subjects had improved (Figure 3).

When single-layer grassland was used as a restorative environment, NA scores in the pre-restoration (P_1_) stage were significantly greater than those in the post-restoration (P_2_) stage [*t* (57) = 4.91, *p* < 0.001, *d* = 1.10, 95% confidence interval (CI) (5.60, 13.90)]. When the tree–grass composite woodland was used as a restorative environment, the NA scores in the pre-restoration (P_1_) stage were significantly greater than those in the post-restoration (P_2_) stage [*t* (57) = 4.96, *p* < 0.001, *d* = 1.09, 95% CI (5.92, 14.58)]. When the tree–shrub–grass composite woodland was used as a restorative environment, the NA scores in the pre-restoration (P_1_) stage were significantly greater than those in the post-restoration (P_2_) stage [*t* (57) = 8.48, *p* < 0.001, *d* = 1.90, 95% CI (9.94, 16.46)]. Finally, when single-layer woodland was used as a restorative environment, NA scores in the pre-restoration (P_1_) stage were significantly greater than those in the post-restoration (P_2_) stage [*t* (57) = 4.88, *p* < 0.001, *d* = 1.09, 95% CI (6.05, 15.15)] (Table 2). The above results show that the NA scores of the subjects were significantly decreased after viewing the photos of the four groups of plant communities.

**Table 2:** Analysis results of paired samples *T*-test and the average NA values of subjects in two stages (P_1_ and P_2_).

Plant communities	M (P_1-NA_)	SE (P_1-NA_)	M (P_2-NA_)	SE (P_2-NA_)	*t*	*p*
Single-layer grassland	21.20	8.95	11.45	1.64	4.91	0.000
Tree-grass composite woodland	22.10	9.11	11.85	2.52	4.96	0.000
Tree-shrub-grass composite woodland	24.75	6.77	11.55	1.99	8.48	0.000
Single-layer woodland	22.90	9.74	12.30	2.54	4.88	0.000

*p < 0.05, **p < 0.01, ***p < 0.001. *N* = 20 for each group.

When single-layer grassland was used as a restorative environment, the PA scores in the pre-restoration (P_1_) stage were significantly lower than those in the post-restoration (P_2_) stage [*t* (57) = −2.49, *p* < 0.050, *d* = −2.49, 95% CI (−9.29, −0.81)]. When the tree–grass composite woodland was used as the restorative environment, the PA scores in the pre-restoration (P_1_) stage were significantly lower than those in the post-restoration (P_2_) stage [*t* (57) = −2.78, *p* < 0.050, *d* = −2.78, 95% CI (−9.91, −1.39)]. When the tree–shrub–grass composite woodland was used as the restorative environment, the PA scores in the pre-restoration (P_1_) stage were significantly lower than those in the post-restoration (P_2_) stage [*t* (57) = −2.34, *p* < 0.050, *d* = −2.34, 95% CI (−8.06, −0.44)]. When single-layer woodland was used as a restorative environment, the PA scores in the pre-restoration (P_1_) stage were significantly lower than those in the post-restoration (P_2_) stage [*t* (57) = −3.06, *p* < 0.050, *d* = −3.06, 95% CI (−9.43, −1.77)] (Table 3). It can be seen that the PA scores of the subjects increased significantly after viewing the photos of the four groups of plant communities. In summary, viewing the photos of four groups of plant communities has a significant effect on emotional recovery, and each type of plant community has a very significant effect on negative emotional recovery and a significant effect on positive emotional recovery.

**Table 3:** Analysis results of paired samples *T*-test and average PA values of subjects in two stages (P_1_ and P_2_).

Plant communities	M (P_1-PA_)	SE (P_1-PA_)	M (P_2-PA_)	SE (P_2-PA_)	*t*	*p*
Single-layer grassland	21.90	8.49	26.95	7.71	−2.49	0.022
Tree-grass composite woodland	20.75	7.48	26.40	6.98	−2.78	0.012
Tree-shrub-grass composite woodland	22.45	8.65	26.70	7.42	−2.34	0.031
Single-layer woodland	20.80	6.45	26.40	7.37	−3.06	0.006

*p < 0.05, **p < 0.01, ***p < 0.001. *N* = 20 for each group.

#### 4.1.2 Emotional recovery effects of plant communities

**Figure 4:**
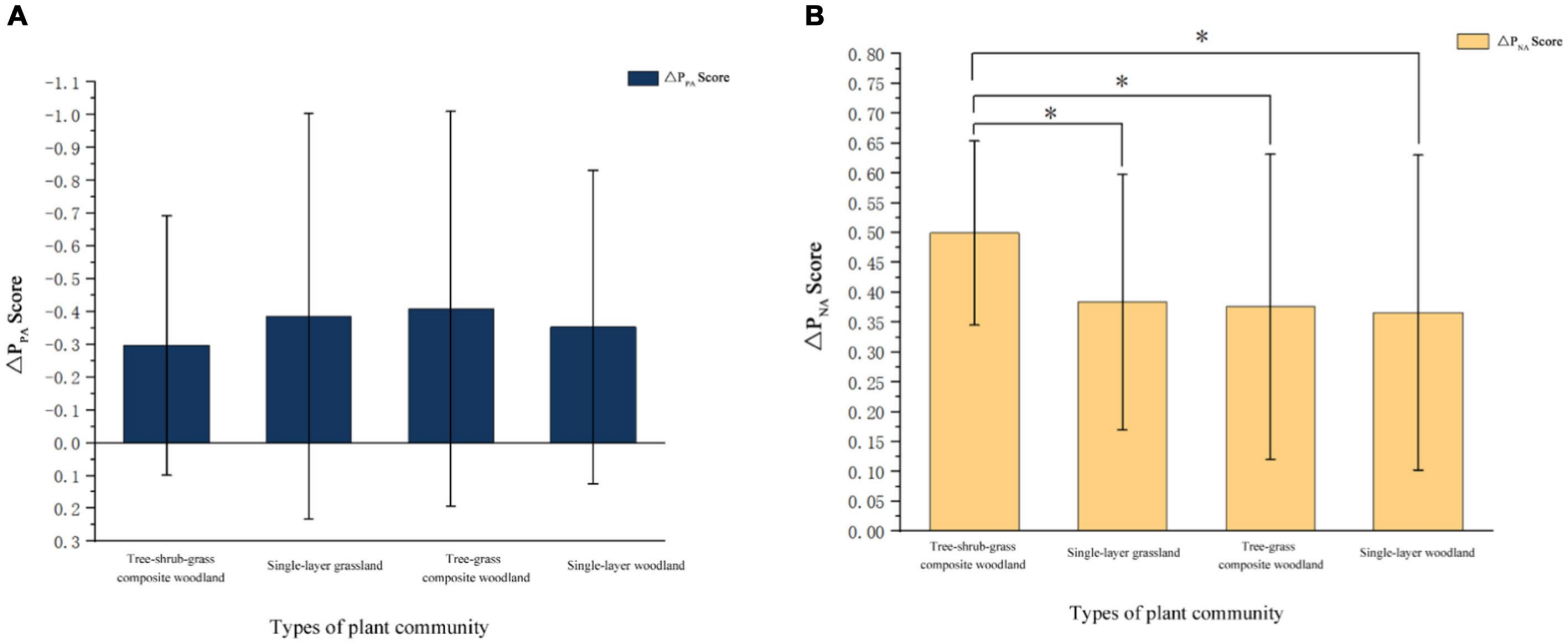
Comparison of landscape scores of four groups of plant communities (*p < 0.050); Data is expressed as a *M*, *N* = 20 for each group.

These results indicate that each group of plant communities can promote the recovery of negative emotions. In order to further confirm whether there were differences in the emotional recovery effect among the four groups of plant communities, the negative and positive emotional change values (ΔP_NA_, ΔP_PA_) were calculated according to Part 3 as indicators of the emotional recovery effect. Then, the effect of emotional recovery was tested by repeated-measures analysis of variance. According to the results of repeated-measures analysis of variance, significant differences existed in the impact of the four groups of plant-community landscapes on negative-emotion recovery (Figure 4), but no significant differences could be found in the impact on positive-emotion recovery. The recovery effect of negative emotion scores in the tree–shrub–grass composite woodland was significantly greater than that in single-layer grassland (*p* = 0.008), tree–grass composite woodland (*p* = 0.016), and single-layer woodland (*p* = 0.023), respectively. In conclusion, among the four groups of plant communities, the tree–shrub–grass composite woodland displayed the most significant effect on the negative emotion relief of the subjects.

### 4.2 EEG analysis results

**Figure 5:**
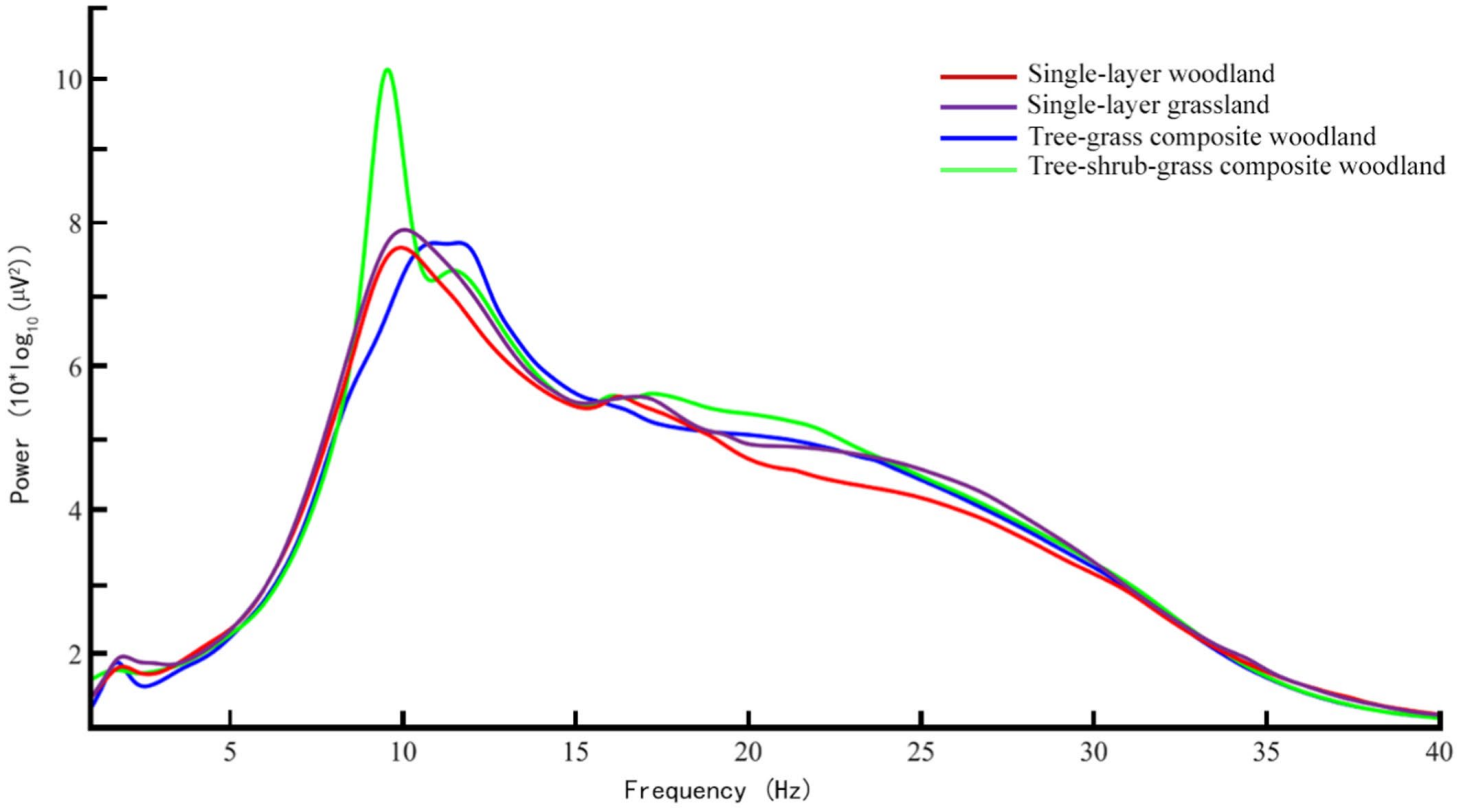
The average spectral map of four groups of plant community landscape, *N* = 20 for each group.

**Figure 6:**
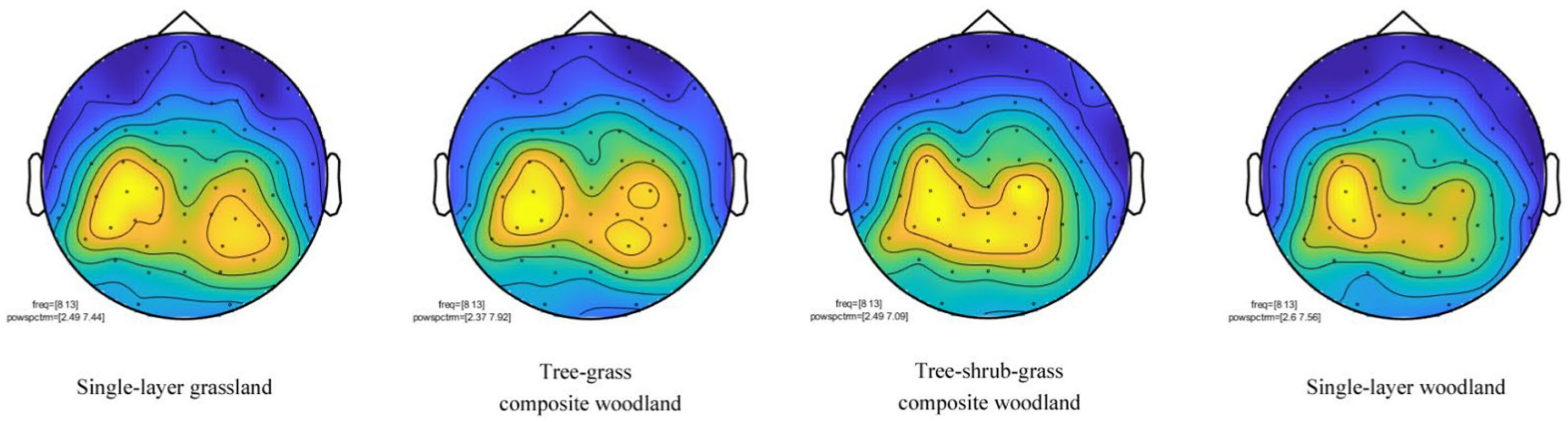
*α* wave brain topographic map of plant community landscape in four groups, *N* = 20 for each group.

After the EEG analysis of single subjects was completed, the EEG data of 20 subjects were analyzed as a whole, and the average spectral map of four electrode points (P1, PZ, PO3, OZ) (Figure 5) and the EEG topographic map of alpha waves (Figure 6) were obtained.

It can be seen from Figure 4 that the spectrum peak points of alpha waves in the four groups of plant communities were higher than the spectrum peak points of the other three brain waves. Among them, the alpha-wave energy values (8–13 Hz) of the tree–shrub–grass composite woodland were significantly higher than those of the three plant-community landscapes of single-layer grassland, tree–grass composite woodland, and single-layer woodland, respectively. Repeated-measures analysis of variance was performed on the alpha-wave energy values of the four groups, and it was found that there were significant differences between the alpha-wave energy values of the four groups of plant communities (Table 4). The results showed that the alpha-wave energy values of the tree–shrub–grass composite woodland community were significantly greater than those of the tree–grass composite woodland [*t* (57) = 9.50, *p* < 0.010, *d* = 0.46, 95% CI (0.46, 0.68)], single-layer woodland [*t* (57) =16.75, *p* < 0.010, *d* = 0.03, 95% CI (0.59, 0.74)], and single-layer grassland [*t* (57) = 9.50, *p* < 0.010. *d* = 0.31, 95% CI (0.30, 0.45)] plant communities.

**Table 4:** Results of α wave energy analysis of four groups of plant communities.

Plant communities	*M*	SD	*F*	*p*	*η*^2^
Single-layer grassland	7.13	0.63	139.05	0.000	0.218
Tree-grass composite woodland	6.94	0.79			
Tree-shrub-grass composite woodland	7.50	1.24			
Single-layer woodland	6.84	0.62			

*p < 0.050, **p < 0.010, ***p < 0.001. *N* = 20 for each group.

In a topographic map of the brain, yellow indicates electrical activity, blue indicates inactivity, and alpha waves are distributed at the top of the brain. Figure 5 shows the brain electrical activity at the top, indicating that the subject was in an awake and relaxed state during visual perception; notably, the bright yellow area when viewing the tree–shrub–grass composite woodland appeared larger than that observed when viewing the other three groups of plant communities, indicating that the subject was most awake and relaxed during visual perception of the former. This shows that participants did feel happy and relaxed when viewing the four groups of plant community photos, particularly the photos of tree–shrub–grass composite woodland, and negative emotions such as tension, stress, and anxiety were reduced.

## 5 Discussion

The objective of this study was to investigate the effectiveness of plant communities in alleviating negative emotions of college students through visual perception and to examine potential differences in the recovery effects of viewing different plant communities. First, the PANAS results demonstrated that viewing photos of plant communities can enhance the positive emotions and reduce the negative emotions of college students. Furthermore, distinct plant communities exhibited significant effects on emotional recovery, with the tree–shrub–grass composite woodland demonstrating the most pronounced effect on alleviating college students’ negative emotions. Second, further analysis of the EEG data showed that visual perception of tree–shrub–grass composite woodland was best for the emotional recovery of college students. The objective findings from EEG analysis were consistent with the subjective outcomes of the questionnaire, indicating that, irrespective of subjective perception or objective description, the degree of emotional recovery was significantly greater after viewing tree–shrub–grass composite woodland compared to after viewing single-layer grassland, tree–grass composite woodland, or single-layer woodland.

### 5.1 Effects of plant communities on emotional recovery

During visual perception, the negative emotion scores of the subjects decreased, which may be attributed to the fact that green color can effectively reduce pressure, relieve fatigue, and promote a positive psychological response (Gu et al., 2012). Humans exhibit a preference for green plants over plants of other colors (Elsadek, 2014). Furthermore, natural landscapes have been shown to improve negative emotions. Contact with plants increases alpha waves, reduces stress (Hassan, 2018), and induces relaxation (Jeong and Park, 2021). As the number of plants increases, negative emotions gradually decrease, and the low-frequency band of the EEG relaxation band increases (Jung et al., 2023). Previous research has also indicated that viewing nature-related landscape elements, such as bamboo forests (Hassan et al., 2018) and lawns (Wang, 2019), is positively correlated with emotional restoration. Therefore, viewing photos of plant communities provided participants with an opportunity to recover their emotions, which is consistent with previous findings (Russell and Mehrabian, 1978; Ulrich et al., 1991).

Previous studies have suggested that viewing single-layer grassland has the best restoration effect (Balling and Falk, 1982), which contrasts with the results of this study. However, other studies have demonstrated that the restoration effect of viewing single-layer grassland is low (Sonntag-Öström et al., 2015), consistent with the findings of this study. These differences may be due to variations in objective factors, such as plant species, plant growth, and planting methods, in each study, resulting in different research outcomes. In addition, previous research has also shown that the restoration effect of the same landscape component can differ due to different spatial organization (Parsons, 1995). Hence, the experimental results substantiate the initial conclusion that various plant communities have distinct impacts on human emotional recovery. The shapes of all plant species help improve vision. For example, trees can divide space, and shrubs can anchor structures to the ground, grass helps define the edges of space (Smardon, 1988). This disparity may arise from the diverse spatial structures engendered by different plant communities and the abundance of plant species (Han, 2007; Tarashkar et al., 2019).

### 5.2 Reasons why tree–shrub–grass composite woodland facilitates emotional recovery better than other plant communities

Psychological research shows that stress is a psychological manifestation when environmental demands exceed personal psychological resources, and people will actively seek out a sense of security to reduce their stress (Lambert and Lazarus, 1970). Habitat theory contends that the relationship between people and the natural world can be attributed to an interdependent process. Human beings’ love for scenery stems from the fact that the aesthetically fulfilled subject lives in an environment that meets their survival needs—that is, when an individual is in an environment that meets their own survival needs, they will appreciate it. Since organisms tend to be aesthetically inclined to survive, people’s aesthetic preferences for their environment ultimately serve to improve their quality of life. Good environmental features can stimulate people’s intuitive positive reactions. If environmental conditions are considered conducive to survival, a person will experience a positive emotional response; conversely, if conditions are unlivable, they experience an anxious or uneasy response. Landscape features can affect people’s psychological state and physiological activities and subsequently dictate their emotional changes. As such, people can judge the quality of the environment by evaluating whether landscape features promote human survival. For example, people’s love for plant flowers comes from the forthcoming abundancy of fruits predicted by the large number of flowers, which can meet human food needs. In addition, people’s pursuit of the beauty of their cultural heritage often comes from the appearance of certain relics, whose presence also suggests the historical favorability of the environment for human life.

Prospect–refuge theory explains that humans prefer natural environmental conditions with lookout and refuge characteristics. The so-called lookout characteristic refers to the fact that an individual can extend their sight into the scenery without being obscured, and it also allows people to observe, understand, and control their surrounding objects. Lookout is an attribute of the environment, providing an unobstructed view for the individual, thereby giving the ability to locate distant resources and identify various dangers that may be encountered on the road. Meanwhile, the so-called shelter characteristic means that the landscape provides actual or imagined escape opportunities for individuals to avoid potential dangers, such as other creatures and environments (storms, cliffs, etc.). Therefore, a landscape that can be seen but not seen can give people a sense of security, a sense of relaxation, and a sense of pleasure.

According to the above two theories, we can see that, among the four plant types involved in this experiment, tree-shrub–grass composite woodland has both lookout and shelter characteristics (Moscoso et al., 2015). Although the single-layer grassland has strong observation characteristics and unobstructed vision, it does not have the characteristics of shelter. Not only can the subjects not hide in it, but their sense of security is also insufficient; thus, it is not adequate to attract strong subject attention. Single-layer woodland has strong protective properties and is easy for subjects to hide in; however, due to obstructing their line of sight, its observation performance is very low, and subjects cannot predict threats they may face, so the sense of security is also insufficient. Finally, the protection functions of tree-grass composite woodland and single-layer grassland are also low, and the viewing performance associated with these communities is not as good as that associated with single-layer grassland.

Among the four plant community types, tree–shrub–grass composite woodland has the best effect. First, the activity space in front of it (within 5–10 m) is a patch of grass without obstruction; although the viewing characteristics associated with this community type are not as good as those affiliated with single-layer grassland, it still has strong lookout characteristics. Second, the existence of shrubs forms a space for subjects to hide such that any animal threats outside this area would not see them. At the same time, the plant species of the tree–shrub–grass composite woodland are rich and diverse, and the trees with high trunk branches, dense canopy, and moderate leaf length, which have a higher priority (Zhao et al., 2017), can better attracting the subjects’ curiosity about the environment; these aspects together meet the needs of college students for private space (Kaplan, 1995). Especially in the urban environment, where the pressure is high and residents are isolated from nature for long periods of time, college students are extremely eager to escape such a noisy environment for a quieter, natural one. At the same time, the plant community structure of tree–shrub–grass composite woodland is complex, spatially secretive, and supported by vegetation (Sonntag-Öström et al., 2015). Therefore, in this plant community environment, the subjects and the natural environment reach an optimal state of comforting coexistence (Gatersleben and Andrews, 2013), so the former can recover from stress and fatigue faster.

The results of this study have important implications for future plant community planning and design. Studying the interaction between college students and different plant communities can help promote contact between college students and the natural environment, alleviate negative emotions caused by social factors, and improve self-happiness while, at the same time, promoting tree–shrub–grass composite woodland with good mood recovery function and other plant community construction. In subsequent research, the optimal plant configuration of the tree–shrub–grass composite woodland plant community can be planned in further detail; this might involve the detailed selection of tree species, as different plant species have unique textures, shapes, and colors. These aspects, combined with the characteristics of the plants themselves, can give full play to the healing function of the tree–shrub–grass composite woodland.

### 5.3 Limitations

At present, this study has several limitations that should be acknowledged. First, in the selection of photos for this experiment, efforts were made to minimize variations between the plant community groups; for instance, it was ensured that plant leaves were uniformly green, without other colors. However, due to factors like diverse plant species and community morphology, there may still be inherent errors in the experimental results. Additionally, the sample size of this study was small, with a population consisting solely of college students. Therefore, future research should aim to recruit a larger and more diverse sample to analyze specific objective and subjective changes in visual perception.

Second, due to the experimental design, this study only measured positive and negative affect scores under acute stress and did not capture long-term cognitive indicators. Future studies should consider incorporating measures that assess cognitive functioning over an extended period.

Lastly, this experiment solely examined emotional recovery based on visual perception and did not account for the combined effects of other senses. As technology advances, future research can extend the duration of visual perception and include a broader range of sensory experiences.

## 6 Conclusion

As an integral element of natural landscapes, plants are extensively utilized in park construction. However, the impact of different plant communities on college students’ emotional recovery has received limited attention. Therefore, the objective of this study was to investigate whether plant communities can effectively alleviate negative emotions in college students through visual perception alone and whether there are variations in the recovery effects among different plant communities. To ensure data accuracy, this study employed a combination of subjective evaluation and objective EEG measurements to examine the influence of four distinct plant community types on college students’ emotional recovery.

One of the key findings of this study was that, under a single visual perception, all four plant community types demonstrated positive effects on emotional recovery, albeit to varying degrees. This suggests that, in future plant community planning, the configuration of plant communities can be flexibly designed based on college students’ needs and site conditions. For instance, a composite woodland consisting of trees, shrubs, and grass can offer privacy, while a tree–grass composite woodland can provide a space for leisure activities. Additionally, a single-layer woodland can provide shade, and a single-layer grassland can serve as a venue for various activities. By aligning the planning for emotional recovery with the specific requirements of individuals and plant communities, it is possible to enhance college students’ engagement with nature and improve their self-happiness, thereby effectively alleviating their stress, anxiety, and other negative emotions and promoting their overall physical and mental well-being.

## Data availability statement

The original contributions presented in the study are included in the article/supplementary material, further inquiries can be directed to the corresponding authors.

## Ethics statement

The studies involving human participants were reviewed and approved by Suzhou University of Science and Technology Ethics Committee. The patients/participants provided their written informed consent to participate in this study.

## Author contributions

WJF: Data curation, Investigation, Software, Visualization, Writing – original draft. FG: Conceptualization, Methodology, Writing – review & editing. XZ: Conceptualization, Project administration, Writing – review & editing. BD: Conceptualization, Methodology, Writing – review & editing. XLC: Investigation, Writing – review & editing. XX: Investigation, Writing – review & editing. ZYY: Investigation, Writing – review & editing. YL: Investigation, Writing – review & editing.
