# Corrigendum: Tree–shrub–grass composite woodland better facilitates emotional recovery in college students emotion better than other plant communities

**DOI:** 10.3389/fpsyg.2024.1386406

**Published:** 2024-04-09

**Authors:** Wen Jun Fu, Fei Gao, Xing Zhang, Bo Dong, Xi Lin Chen, Xin Xu, Zhi Yu Yang, Yong Liu

**Affiliations:** ^1^School of Architecture and Urban Planning, Suzhou University of Science and Technology, Suzhou, Jiangsu, China; ^2^School of Education, Suzhou University of Science and Technology, Suzhou, Jiangsu, China; ^3^Suqian High Speed Railway Construction and Development Co., Ltd., Suqian, Jiangsu, China

**Keywords:** plant community, restorative landscape, positive emotions, negative emotions, EEG

In the published article, there was an error. One of the plant names in the paper was misspelled. A correction has been made to *2. Materials and methods, 2.1. Stimulus materials, Paragraph 1*.

This sentence previously stated:

Consequently, the plant communities studied in this experiment included single-layer grassland (*Festuca elata* Keng ex E. B. Alexeev), single-layer woodland [*Taxodium distichum* (L.) Rich.], tree–grass composite woodland [*Osmanthus fragrans* (Thunb.) Lour. and *Festuca elata* Keng ex E. B. Alexeev], and tree–shrub–grass composite woodland [*Cinnamomum camphora* (L.) J. Presl, *Rhododendron* × *pulchrum* Sweet and *Festuca elata* Keng ex E. B. Alexeev].

The corrected sentence appears below:

Consequently, the plant communities studied in this experiment included single-layer grassland (*Festuca elata* Keng ex E. B. Alexeev), single-layer woodland [*Taxodium distichum* (L.) Rich.], tree–grass composite woodland [*Osmanthus fragrans* (Thunb.) Lour. and *Festuca elata* Keng ex E. B. Alexeev], and tree–shrub–grass composite woodland [*Celtis sinensis* Pers., *Rhododendron* × *pulchrum* Sweet and *Festuca elata* Keng ex E. B. Alexeev].

The authors apologize for this error and state that this does not change the scientific conclusions of the article in any way. The original article has been updated.

