# Erratum: Tree–shrub–grass composite woodland better facilitates emotional recovery in college students emotion better than other plant communities

**DOI:** 10.3389/fpsyg.2024.1425682

**Published:** 2024-05-14

**Authors:** Frontiers Production Office

**Affiliations:** Frontiers Media SA, Lausanne, Switzerland

**Keywords:** plant community, restorative landscape, positive emotions, negative emotions, EEG

Due to a production error, co-first authorship was not clearly denoted for authors “Wen Jun Fu” and “Fei Gao.” These authors contributed equally to this work and share first authorship. The corrected author details are shown below.

Wen Jun Fu^1†^, Fei Gao^1†^, Xing Zhang^1*^, Bo Dong^2*^, Xi Lin Chen^3^, Xin Xu^1^, Zhi Yu Yang^1^ and Yong Liu^1^

^1^School of Architecture and Urban Planning, Suzhou University of Science and Technology, Suzhou, Jiangsu, China

^2^School of Education, Suzhou University of Science and Technology, Suzhou, Jiangsu, China

^3^Suqian High Speed Railway Construction and Development Co., Ltd., Suqian, Jiangsu, China

^*^Correspondence:

Xing Zhang

2605@usts.edu.cn

Bo Dong

dongb283@126.com

^†^These authors have contributed equally to this work and share first authorship

The publisher apologizes for this mistake. The original article and the corrigendum published on 09 April 2024 have been updated.

